# Effect of Sugar Replacement with Stevia-Based Tabletop Sweetener on Weight and Cardiometabolic Health among Indian Adults

**DOI:** 10.3390/nu15071744

**Published:** 2023-04-03

**Authors:** Govindarajan Raghavan, Arohi Bapna, Arti Mehta, Akash Shah, Tejas Vyas

**Affiliations:** Zydus Wellness Institute, Zydus Wellness Products Limited, R & D Centre, Plot No. 115/5, TP Scheme No. 51, Near the Bliss & North One Apartment, Off Ambli-Bopal Road, Ahmedabad 380058, India

**Keywords:** stevia, low-calorie sweetener, weight, obesity, prediabetes, steviol glycosides, overweight

## Abstract

Added sugars contribute to caloric intake in the diet, which may lead to conditions like obesity. Replacing added sugar with a natural sugar substitute like zero-calorie stevia may help in reducing sugar and caloric intake. Methods: An open-label, single-arm pilot study was conducted to evaluate the efficacy and safety of a stevia-based tabletop sweetener among overweight subjects with normal blood sugar levels (*n* = 24) and overweight prediabetic subjects (*n* = 21). Subjects were instructed to replace added sugar in their daily diet with the test product over a study period of 90 days. Primary outcomes included change in body weight and waist circumference, while the secondary outcomes included change in blood glucose (PPBG), body mass index (BMI), and lipid parameters evaluated at baseline, 30 days, 60 days, and 90 days. Glycated hemoglobin (HbA1c) was evaluated at baseline and 90 days. Results: When compared with the baseline, a significant reduction in weight (*p* < 0.001) and waist circumference (*p* < 0.001) was observed at Day 90 in overweight subjects. Similarly, in overweight prediabetic subjects, a significant reduction in weight (*p* < 0.001) and waist circumference (*p* < 0.05) with non-significant change in HbA1c was observed at Day 90 from baseline. In a subgroup analysis, among the subjects who lost weight, 2.12 kg (*n* = 35) weight loss and 4.78 cm (*n* = 32) reduction in waist circumference were observed at 90 days. No adverse outcomes were reported throughout the study period. The consumption of steviol glycosides was within the acceptable daily intake (ADI). Conclusions: Replacing added sugar in the daily diet with stevia-based tabletop sweetener, along with a physical activity regimen, promotes weight loss and reduction in waist circumference in overweight subjects with normal blood sugar levels and prediabetic subjects (CTRI/2019/12/022470).

## 1. Introduction

Obesity is a global health concern. The World Health Organization (WHO) reports that the incidence of obesity has tripled over the last four decades. In 2016, 39% of the adult population globally was overweight, of which 13% were obese. Being overweight or obese causes pathophysiological changes in the human body and increases the risk for comorbidities [1]. Indians today are facing a major public health challenge of non-communicable diseases (NCDs), including diabetes, cardiovascular disorders, and hypertension [2]. Several epidemiological and interventional studies have highlighted the association between sugar consumption and weight gain, leading to an increased risk of cardiovascular disease (CVD) and type 2 diabetes mellitus (T2DM) through fat gain and increased body weight [3].

The International Sugar Association reported that the world sugar consumption rose to 168.479 million tons in 2021, following three years of decline. The major sugar-consuming markets include India, China, the USA, Russia, Brazil, Indonesia, Pakistan, Mexico, and Egypt [4]. The intake of sugar and sweets has been popular in Indian culture and traditions since ancient times. Jaggery, khandsari, sweets, confectionaries, and sugar-sweetened beverages (SSBs) are sources of added sugar in the Indian diet. Reports have suggested an increase in sugar intake from SSBs over the years in India. Surprisingly, the total sugar intake in Indians exceeded the average global per capita consumption when intake from traditional sugars and SSBs was added to that of white sugar [5]. The 66th round of the National Sample Survey Office (NSSO, India) 2009–2010 study reported a 151.36% increase in white sugar consumption per person/day in India from 2000 to 2010. In the year 2000, the amount of white sugar consumed per person/day was 22 g/day, which had increased to approximately 55.3 g/day (13.2 teaspoons) in 2010 [6,7].

Despite the high consumption, white sugar provides empty calories (1 g of sugar gives 4 kcal) and lacks natural minerals. Additionally, the SSBs consumed regularly are high in calories and glycemic load. Therefore, in view of the increased sugar intake and associated adverse health outcomes, the 2011 consensus dietary guideline for Indians, in line with the World Health Organization (WHO, Geneva, Switzerland), recommends reducing the daily intake of free sugars to less than 10% of the total energy intake [5,8]. Apart from limiting free sugar intake, the use of FDA-approved sugar substitutes called non-nutritive sweeteners (NNSs) can be utilized in various foods and beverages. NNSs are sweeter than sugar and contribute negligible calories when added to food [9]. In 2012, the American Heart Association (AHA, Texas, TX, USA) and the American Diabetes Association (ADA, Virginia, NV, USA) reported that NNSs, when used judiciously, may facilitate a reduction in added sugars and energy intake, thereby helping people to achieve and maintain healthy body weight and lower the risk of cardiometabolic diseases [10].

Steviol glycosides, derived from the leaves of *Stevia rebaudiana* Bertoni, are natural, sweet-tasting, calorie-free NNSs that may be used as a sugar substitute [11]. These are reported to have 200–400 times more sweetness than table sugar. High-purity steviol glycosides are generally recognized as safe (GRAS) and are approved by the US FDA and the European Food Safety Authority (EFSA) for human use. The acceptable daily intake (ADI) is the amount of food additive that can be consumed over a lifetime without any health consequences. ADI of steviol glycosides (expressed as steviol) by various regulatory bodies is given as 0–4 mg/kg/day [9]. Clinical evidence suggests that steviol glycosides have positive effects on insulin sensitivity and postprandial glucose levels in humans with diabetes mellitus [11,12]. However, limited evidence is available on the effect of steviol glycosides on weight reduction in human subjects [13], especially in the Indian population. An open-label, single-arm pilot clinical study was conducted to evaluate the efficacy and safety of stevia-based tabletop sweetener formulation when used to replace added sugar in the diet among overweight healthy subjects with normal sugar levels and overweight prediabetic subjects under a physical activity regimen.

## 2. Materials and Methods

### 2.1. Test Product

Stevia-based tabletop sweetener was used as the test product, which was available in two forms–granules and pellets. Steviol glycosides were purchased from PureCircle India. It was dry mixed with polyol (maltitol) and other allowed carriers and granulated into a free-flowing powder referred to as powder concentrate. Pellets were also prepared using steviol glycosides in a standard tablet preparation process with permitted excipients. The sweetness of the test product was defined as 1 g of powder concentrate = 1 teaspoon (5 g) of table sugar in sweetness and 1 pellet (100 mg) = 1 teaspoon (5 g) of table sugar in sweetness.

#### Quantification of Steviol Glycosides

High-performance liquid chromatography (HPLC) analysis was performed to calculate the amount of steviol glycosides in the test product [14]. Shimadzu (Kyoto, Japan) HPLC system (Model: LC-2030C 3D, S. No: L21455502297) was used, which consisted of a standard autosampler, thermostat column, vacuum degasser, quaternary pump, and variable wavelength detector. The separation was achieved on a stainless-steel silica-based Phenomenex Luna C18 (4.6 mm × 250 mm, 5 μm) column (S. No: H18-224670). The mobile phase was selected as per the Joint FAO/WHO Expert Committee on Food Additives (JECFA) 2010, with an isocratic solvent system consisting of a mixture (68:32 *v*/*v*) of 10 mmol/L sodium phosphate buffer and acetonitrile having pH 2.6 (pH adjusted with orthophosphoric acid) at 1.0 mL/min flow rate. The absorption was measured at 210 nm for steviol glycoside. The chromatographic data were acquired, monitored, and processed using Shimadzu Lab Solutions software (version 6.82) from Shimadzu Corporation.

### 2.2. Study Design

An open-label, single-arm pilot study was conducted to evaluate the efficacy and safety of the test product among overweight healthy subjects with normal blood sugar levels and overweight prediabetic subjects when used to replace added sugar in the diet. The study was conducted by Cliantha Research, Ahmedabad, Gujarat, India, by following the Indian Council of Medical Research (ICMR, New Delhi, India) ethical guidelines, The International Council for Harmonization of Technical Requirements for Pharmaceuticals for Human Use (ICH) (Step 5) “Guidance on Good Clinical Practice” (E6 R2), and the Declaration of Helsinki. The OM Institutional Ethics Committee approved the study protocol, English and Gujarati informed consent document (ICD), case report form (CRF), and meal menu on 06 December 2019 before the initiation of the investigation. The study was registered with the Clinical Trials Registry—India (CTRI/2019/12/022470).

The study was conducted from 2 January 2020 to 30 December 2020. Due to the COVID-19 outbreak in India, the central government imposed a nationwide lockdown from 25 March 2020 to 31 May 2020. Hence, the enrolled subjects were not able to report at the facility for their respective scheduled study visits. The interim study–lab data of scheduled study visits was thus missing for a few subjects. During this period, the test product was provided at their home by the study personnel and couriered to a few enrolled subjects with appropriate documentation. Telephonic follow-ups were conducted to evaluate compliance and safety concerns of the test product.

### 2.3. Study Population

Eligible participants were informed in detail about the purpose of the study, and the treatment was fully explained before obtaining informed consent. The study was conducted in two groups: Group 1 consisted of overweight healthy subjects with normal blood sugar levels, and Group 2 consisted of overweight prediabetic subjects.

#### 2.3.1. Inclusion Criteria

Subjects were included in the study if they met the following criteria:

- Age between 18 to 55 years (inclusive), overweight subjects with body mass index (BMI) of ≥25 to ≤30.0 kg/m^2^, prediabetic subjects with fasting blood glucose level of 100–125 mg/dL and HbA1c levels of ≥5.7% and ≤6.4%, and subjects with normal renal and liver functions.
- Subjects willing to follow a diet plan, subjects willing to reduce their weight and waist circumference, and subjects with a history of stable weight defined as no significant weight change (less than ±5%) in the three months before enrollment and physical activity level lesser than 2 h/day.
- Subjects willing to abstain from eating any other sugar-containing products or any other form of sugar usage during the study duration.

#### 2.3.2. Exclusion Criteria

Subjects were not enrolled in the study if they met at least one of the following criteria: pregnant females or females planning to get pregnant during the study duration, subjects having type 1 or type 2 diabetes mellitus, subjects with insulin resistance, subjects with lipid abnormalities, subjects diagnosed with eating disorders such as bulimia or binge eating, history of positive HIV test, history of hepatitis B infection, subjects with any history of drug and alcohol abuse, history of cancer, or history of clinically significant cardiac disease or endocrine abnormalities.

### 2.4. Study Protocol

The subjects were given the powder concentrate form and pellet form of the test product to replace the added sugar intake in the diet to be used over the entire 90 days of the study period. No other form of sugar was permitted during the study. The subjects had dietary restrictions of 2300 kcal/day in women and 2500 kcal/day in men, with moderate exercise determined by 45 min of brisk walking or gym exercises daily. The potential subjects were screened as per the inclusion and exclusion criteria after obtaining written informed consent. At Visit 01, screening was performed as per the inclusion and exclusion criteria, and the subjects were informed about the next visit. At Visit 02, the subjects were enrolled for the study after the verification as per inclusion and exclusion criteria. The subjects visited the facility on Day 30, Day 60, and Day 90 for all the laboratory investigations.

### 2.5. Study Outcomes

#### 2.5.1. Primary Outcomes

The effect of the test product was measured in terms of change in body weight from baseline, i.e., Day 01 to Day 30, Day 60, and Day 90. The effect of the test product was measured in terms of change in waist circumference from the baseline, i.e., Day 01 to Day 30, Day 60, and Day 90.

#### 2.5.2. Secondary Outcomes

The effect of the test product was assessed in terms of change in mean percentage in postprandial blood glucose (PPBG) level, lipid profile along with lipoprotein levels, and BMI from baseline, i.e., Day 01 to Day 30, Day 60, and Day 90. The change in HbA1c was measured at baseline, i.e., Day 01 and Day 90. The amount of sugar consumption by subjects was also recorded at baseline, i.e., Day 01 and Day 90. Subjective perception assessment was done by the staff on Day 30, Day 60, and Day 90, which included the following questions: “Do you feel lighter than before, after taking this product?” and “Do you feel that there is a change in your waist circumference after using this product?” The safety of the test product was evaluated in terms of the adverse events reported by the subjects or assessed by the investigator throughout the study duration.

### 2.6. Statistical Analysis

Primary and secondary endpoints were evaluated by appropriate statistical tests at a 5% level of significance. Continuous variables like anthropometry and biochemical parameters were described by descriptive statistics (mean and standard deviation). Categorical variables were expressed by frequency and percentage. The statistical analysis was done using SAS^®^ statistical software (Version: 9.4; SAS Institute Inc., Cary, NC, USA). The subjects withdrawn during the study period were not included in the statistical analysis.

## 3. Results

### 3.1. Quantification of Steviol Glycosides

HPLC analysis revealed that stevia-based powder formulation and pellet contained 2.19% *w*/*w* and 20.51% *w*/*w* of steviol glycosides, respectively.

### 3.2. Patient Characteristics

**Figure 1:**
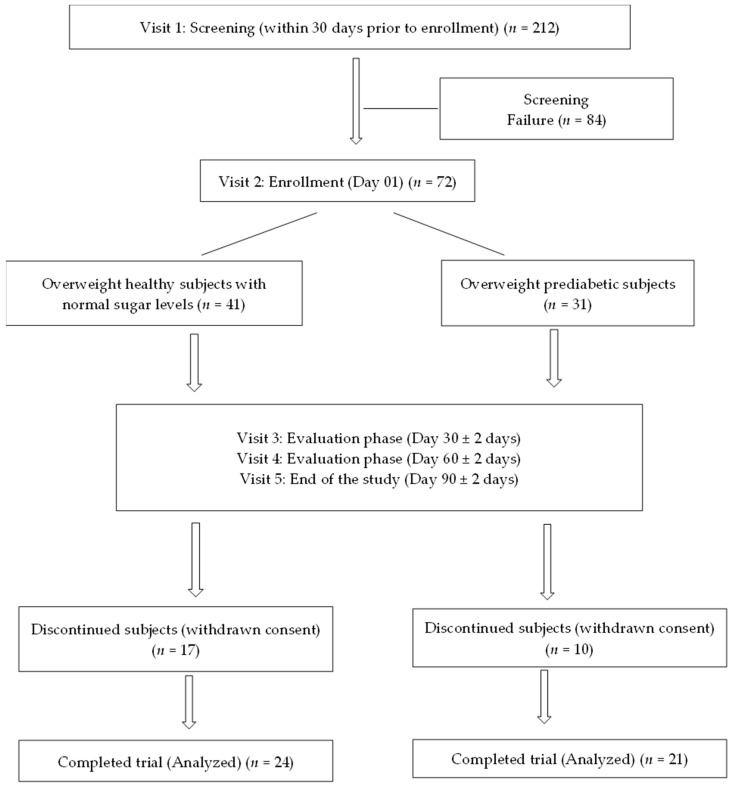
Screening, enrollment, and follow-up of study participants.

A total of 72 subjects (overweight healthy: 41 and overweight prediabetic: 31) were enrolled in this study, and 45 subjects completed the study. The data are missing for a few subjects due to the COVID-19 lockdown imposed during the scheduled study visits. Screening, enrollment, and follow-up data of the subjects are shown in Figure 1.

### 3.3. Primary Outcomes

#### 3.3.1. Changes in Body Weight

In Group 1, a statistically significant decrease was observed in body weight from baseline at Day 90 (*p* < 0.001), while no significant difference was observed from baseline at Day 30 and Day 60 (Table 1).

In Group 2, a statistically significant decrease was observed in body weight from baseline at Day 60 (*p* < 0.05) and Day 90 (*p* < 0.001), whereas no significant difference was observed from baseline at Day 30 (Table 2).

**Figure 2:**
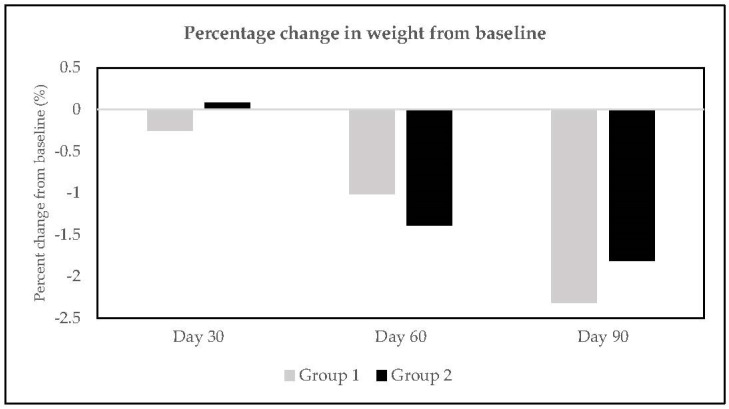
Percentage change in body weight at Day 30, Day 60, and Day 90.

Replacement of added sugar with stevia-based sweetener for 90 days resulted in a significant mean weight loss of 1.63 kg and 1.34 kg in Group 1 and Group 2 subjects, respectively (Table 1 and Table 2). The percentage change in weight from baseline at all visits in both groups is depicted in Figure 2.

Additionally, subgroup analysis revealed that replacing sugar with stevia-based sweetener led to a mean weight reduction of 2.12 kg in 77.77% (i.e., 35 subjects) of the total overweight subjects. This indicates that the replacement of added sugar with stevia-based sweetener reduces weight in overweight subjects when consumed for 90 days.

#### 3.3.2. Changes in Waist Circumference

**Figure 3:**
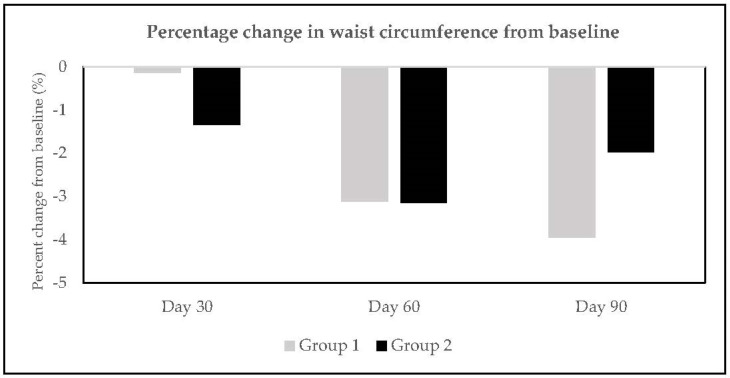
Percentage change in waist circumference at Day 30, Day 60, and Day 90.

In Group 1, a statistically significant reduction was observed in waist circumference at Day 90 (*p* < 0.001); however, no significant difference was observed from baseline at Day 30 and Day 60 (Table 1). In Group 2, a statistically significant reduction was observed in waist circumference from baseline at Day 60 (*p* < 0.05) and Day 90 (*p* < 0.05), while no significant difference was observed from baseline at Day 30 (Table 2). Intake of the test product for 90 days resulted in a significant mean reduction of waist circumference by 1.49 inches (3.79 cm) and 0.75 inches (1.91 cm) in Group 1 and Group 2, respectively (Table 1 and Table 2). The percent change in waist circumference from baseline at all visits in both groups is depicted in Figure 3.

Additionally, the subgroup analysis revealed that replacing sugar with stevia-based sweetener led to a mean reduction in waist circumference of 1.88 inches (4.78 cm) in 71.11% (i.e., 32 subjects) of the total overweight subjects. Sugar replacement with stevia-based sweeteners reduces waist circumference in overweight subjects when consumed for 90 days.

### 3.4. Secondary Outcomes

#### 3.4.1. PPBG Level

In Group 1, a statistically significant difference was observed in PPBG level at Day 30 (*p* < 0.05) and Day 60 (*p* < 0.05) as compared to the baseline; however, no significant difference was observed from baseline at Day 90 (Table 1). In Group 2, no statistically significant difference was observed for change in PPBG level at Day 30, Day 60, and Day 90 as compared with baseline (Table 2).

#### 3.4.2. Lipid Parameters

In Group 1, HDL levels decreased marginally, although they remained significant at all visits. Total cholesterol levels decreased slightly from baseline; however, they were statistically non-significant. LDL, VLDL, and triglyceride levels showed a non-significant increase at Day 90 from baseline (Table 1). In Group 2, a slight non-significant increase in HDL level was observed at Day 60 and Day 90 from baseline. Though non-significant, total cholesterol and LDL levels decreased on Day 30 and Day 60 and increased at Day 90 from baseline. VLDL and triglyceride levels decreased at all visits as compared with the baseline (Table 2).

#### 3.4.3. BMI

In Group 1, a statistically significant difference was observed in BMI from baseline at Day 90 (*p* < 0.0001), while no significant difference was observed from baseline at Day 30 and Day 60 (Table 1). In Group 2, a statistically significant difference was observed in BMI from baseline at Day 60 (*p* < 0.05) and Day 90 (*p* < 0.001) (Table 2).

#### 3.4.4. HbA1c

In both the groups, no statistically significant difference was observed in HbA1c from baseline at Day 90 (Table 1 and Table 2). This shows that stevia-based tabletop sweetener does not cause any change in three-month glycemic control, as evidenced by no change in HbA1C.

#### 3.4.5. Subjective Evaluation

In Group 1, the subjective evaluation revealed that 58.33% of the subjects agreed and 37.50% strongly agreed that they felt lighter than before, and 62.5% agreed while 37.50% strongly agreed that after replacing the sugar with stevia-based sweetener, they felt a change in their waist circumference. In Group 2, 90.48% of the subjects agreed, and 9.52% strongly agreed that they felt lighter than before after replacing sugar with a stevia-based sweetener. Additionally, all the subjects (100%) felt a change in waist circumference after using the test product.

In Group 1 and Group 2, the average amount of calories consumed from sugar was 107.67 kcal/day and 106.0 kcal/day, while the average amount of calories consumed from excipients used in the test product was 16.40 kcal/day and 16.70 kcal/day. The calorie deficit by replacing sugar with stevia-based test products was 91.27 kcal/day and 89.30 kcal/day in Group 1 and Group 2, respectively. The estimated daily intake of steviol glycosides was 2.39 mg/kg body weight/day and 2.4 mg/kg body weight/day in Group 1 and Group 2, respectively, which was less and within the acceptable daily intake of 4 mg/kg body weight/day (Table 3).

#### 3.4.6. Safety Evaluation

No adverse events were reported during the study duration.

## 4. Discussion

Weight gain and obesity may result due to an energy imbalance between calories ingested and calories burned [7]. Reports suggest that an intake of 3500 calories adds one pound to body weight. This indicates that an increase or decrease in calorie intake by 500 calories daily will result in a gain or loss of approximately one pound of body weight per week, i.e., 500 calories per day × 7 days = 3500 calories [15]. Therefore, regulating the calorie density of meals reduces energy consumption, which might be one of the effective ways to reduce body weight.

Non-nutritive sweeteners such as steviol glycosides provide no calories when compared to sugar and may lower the daily sugar and calorie intake, thereby promoting weight loss [7]. In the present study, replacing table sugar with a stevia-based tabletop sweetener resulted in a calorie deficit in both the overweight study groups. Additionally, a significant difference in body weight, waist circumference, and BMI was observed at Day 90 when compared to baseline in both the overweight study groups. Subgroup analysis performed on the overweight subjects who had weight reduction revealed that replacement of sugars with stevia-based tabletop sweeteners led to weight loss in 77.77% of the subjects, with a mean weight reduction of 2.12 kg and reduction in waist circumference in 71.11% of the subjects with a mean waist circumference reduction of 1.88 inches. Clinical studies have reported that the use of non-nutritive sweeteners such as stevia may reduce energy intake and body weight [7,16,17,18,19,20,21]. In a systematic review and meta-analysis conducted on sustained intervention studies, the authors reported a reduction in body weight, BMI, and energy intake with the use of low-calorie sweeteners compared to sugar [16]. A randomized, open-label trial evaluated the efficacy and safety of a novel compound containing stevia and sugar blend in normal to mildly overweight subjects (*n* = 60) [7]. The subjects were divided into two groups. The control group received ordinary table sugar, while the intervention group received a sugar blend containing a combination of stevia and sugar. The results revealed that subjects receiving the sugar blend showed a significantly higher weight loss and reduction in waist circumference at 90 days compared with the control group. Additionally, a significant reduction in total cholesterol, triglyceride, LDL cholesterol, and VLDL cholesterol was observed in the intervention group compared with the baseline [7]. In the present study, calorie deficit after switching to the test product might be one of the possible reasons for weight loss and reduction in waist circumference and BMI in both the overweight study groups. It was noted that replacing sugar with steviol glycosides reduced caloric intake by approximately 90 kcal/day (2700 kcal deficit per month) in both groups, resulting in weight loss.

Evaluation of secondary parameters revealed no clinically significant change in PPBG level, total cholesterol, LDL, VLDL, triglyceride, and HbA1c from baseline to Day 90, and all the values were within the normal laboratory range. This was similarly reported in several clinical studies [22,23,24,25,26,27]. A decrease in PPBG level was observed in overweight healthy subjects with normal sugar levels, which was significant at Day 30 and Day 60, although non-significant at Day 90. Anton et al. (2010) evaluated the effect of stevia, sucrose, or aspartame on satiety, food intake, and postprandial glucose and insulin levels in healthy lean (*n* = 19) and obese (*n* = 12) adult subjects [11]. The subjects received preloads containing stevia (290 kcal), aspartame (290 kcal), or sucrose (493 kcal) before lunch and dinner meals on three separate food test days. Post-hoc analysis revealed that postprandial glucose levels were significantly lower with stevia as compared to sucrose and aspartame at 20 min after preload consumption and 30 min after the test lunch meal. Additionally, the postprandial insulin levels were significantly lower with stevia intake as compared to both sucrose and aspartame. Stevia preloads reduced PPBG and insulin levels, suggesting that stevia may assist with glucose regulation [11].

In the present study, VLDL and triglyceride levels showed a non-significant decrease in overweight prediabetic subjects. A recent systematic review and meta-analysis conducted by Anker et al. (2019) critically evaluated the evidence for the effectiveness of steviol glycosides on human health, particularly on biomarkers of type 2 diabetes [22]. The study included seven studies comprising nine randomized control trials (RCTs), with a total of 462 participants with or without diabetes. The results suggested that intake of steviol glycosides caused non-significant reductions in fasting blood glucose, total cholesterol, and HDL levels. A non-significant increase in LDL and triglyceride levels were also observed. No significant effect on HbA1c was noted as compared with the placebo [22].

Intake of the stevia-based test product resulted in a reduction in table sugar (sucrose) consumption. This may be majorly due to the same sweetness experienced by the subjects with the test product minus the calories from sugar. As reported in the subjective perception questionnaire, most of the subjects from both groups felt lighter than before after taking the test product containing steviol glycosides. Additionally, 62.50% of the overweight healthy subjects with normal sugar levels and all the overweight prediabetic subjects felt a change in their waist circumference after using the test product.

The present pilot study has limitations. As the study is an open-label, single-arm trial, there is a possibility that the results could have been influenced by the participants’ behavior. The stevia-based tabletop sweetener has a positive influence on body weight, waist circumference, and BMI; however, the small sample size might cause possible uncertainty in the results due to participants’ personal choices. Additionally, physical activity is a confounding variable and might have influenced weight loss. This necessitates further research with a larger population on the effect of stevia-based tabletop sweeteners on metabolic parameters under a controlled environment. As this study was conducted during the COVID-19 pandemic period, the missing clinical data on Day 30 and Day 60 could have influenced the interim observations. However, Day 90 data had no missing values.

## 5. Conclusions

Replacing table sugar with natural stevia-based tabletop sweetener provides the same sweetness as sugar and better diet compliance. The use of the stevia-based sweetener reduced weight, waist circumference, and BMI after 90 days when used in combination with a balanced diet and moderate physical activity. A mean weight loss of 1.63 kg and 1.34 kg with a mean waist circumference reduction of 3.79 cm and 1.91 cm was observed in the overweight healthy subjects with normal sugar levels and overweight prediabetic subjects, respectively. The product reduced caloric intake by approximately 90 kcal/day, resulting in a derived calorie deficit of 2700 kcal/month. This might help in reducing the anthropometric parameters without any change in the metabolic parameters. The product was well-tolerated and safe without any reported adverse effects. Therefore, stevia-based tabletop sweeteners are an effective alternative to table sugars in people habituated to high sugar intake and aiming to adapt to a healthy lifestyle. This pilot study was conducted to understand the effect of sugar replacement with stevia-based sweeteners on weight loss as a part of an active lifestyle. Replacement of table sugar with a stevia-based sweetener leads to a calorie deficit in the diet. In a weight loss program, it is important to create an energy deficit, which can be achieved through exercise and reducing caloric intake in the diet. Larger studies with a control group are needed to further validate the results.

## Figures and Tables

**Table 1:** Anthropometric and biochemical characteristics of participants at baseline, Day 30, Day 60, and Day 90 in overweight healthy subjects with normal blood sugar levels.

	Baseline	Day 30	Day 60	Day 90	Change from Baseline ^1^
	(*n* = 24)	(*n* = 24)	(*n* = 11)	(*n* = 24)	(*n* = 24)
Weight (kg)	70.29 ± 8.15	70.11 ± 8.59	66.28 ± 8.27	68.67 ± 8.08 ^†^	−1.63 ± 1.74
Waist circumference (cm)	96.13 ± 4.88	96 ± 5.49	92.64 ± 6.99	92.33 ± 5.51 ^†^	−3.79 ± 4.03
BMI (kg/m^2^)	27.60 ± 1.75	27.48 ± 2.07	27.16 ± 1.78	26.92 ± 2.04 ^ǂ^	−0.67 ± 0.58
PPBG (mg/dL)	114.40 ± 23.44	99.02 ± 18.53 *	95.13 ± 14.80 *	106.81 ± 16.96	−7.59 ± 21.89
Total cholesterol (mg/dL)	166.96 ± 24.04	165.87 ± 24.59	156.72 ± 26.69	166.58 ± 27.02	−0.39 ± 25.09
HDL cholesterol (mg/dL)	41.33 ± 6.61	38.30 ± 5.21 *	37.85 ± 3.87 ^†^	37.81 ± 5.70 *	−3.52 ± 5.83
LDL cholesterol (mg/dL)	103.33 ± 21.49	104.98 ± 20.57	96.99 ± 20.70	105.18 ± 23.01	1.84 ± 21.74
VLDL cholesterol (mg/dL)	22.31 ± 10.61	22.59 ± 11.52	21.88 ± 10.77	23.64 ± 11.55	1.34 ± 8.43
Triglyceride (mg/dL)	111.53 ± 53.07	112.94 ± 57.59	109.41 ± 53.84	118.21 ± 57.74	6.69 ± 42.15
HbA1c (%)	5.43 ± 0.29	-	-	5.50 ± 0.29	0.073 ± 0.32

^1^ Change from baseline to Day 90. Statistically significant values from baseline * *p* < 0.05, ^†^ *p* < 0.001, ^ǂ^ *p* < 0.0001. Values are represented as mean ± standard deviation. BMI: Body mass index; PPBG: Postprandial blood glucose; HDL: High-density lipoproteins; LDL: Low-density lipoproteins; VLDL: Very low-density lipoproteins; HbA1c: Glycated hemoglobin.

**Table 2:** Anthropometric and biochemical characteristics of participants at baseline, Day 30, Day 60, and Day 90 in overweight prediabetic subjects.

	Baseline	Day 30	Day 60	Day 90	Change from Baseline ^1^
	*(n* = 21)	(*n* = 20)	(*n* = 19)	(*n* = 21)	(*n* = 21)
Weight (kg)	73.94 ± 7.03	73.98 ± 7.14	72.84 ± 6.93 *	72.6 ± 6.96 ^†^	−1.34 ± 1.54
Waist circumference (cm)	96.95 ± 5.51	95.65 ± 6.00	93.89 ± 6.98 *	95.05 ± 6.67 *	−1.91 ± 3.58
BMI (kg/m^2^)	27.45 ± 1.66	27.39 ± 1.78	27.09 ± 1.62 *	26.94 ± 1.82 ^†^	−0.52 ± 0.54
PPBG (mg/dL)	106.15 ± 29.84	108.89 ± 29.74	106.37 ± 29.90	115.77 ± 35.56	9.61 ± 34.66
Total cholesterol (mg/dL)	159.02 ± 19.58	152.95 ± 24.51	156.76 ± 25.70	164.00 ± 25.86	4.99 ± 16.30
HDL cholesterol (mg/dL)	36.21 ± 6.14	35.52 ± 6.61	36.85 ± 8.71	37.01 ± 7.64	0.80 ± 6.88
LDL cholesterol (mg/dL)	98.33 ± 16.17	93.51 ± 20.64	96.61 ± 21.83	103.24 ± 22.68	4.91 ± 11.40
VLDL cholesterol (mg/dL)	24.45 ± 10.20	23.92 ± 11.64	23.31 ± 11.67	23.75 ± 12.12	−0.699 ± 8.41
Triglyceride (mg/dL)	122.26 ± 47.68	119.59 ± 47.16	116.54 ± 50.81	118.77 ± 50.41	−3.492 ± 42.05
HbA1c (%)	5.763 ± 0.23	-	-	5.874 ± 0.53	0.111 ± 0.49

^1^ Change from baseline to Day 90. Statistically significant values from baseline * *p* < 0.05, ^†^ *p* < 0.001. Values are represented as mean ± standard deviation. BMI: Body mass index; PPBG: Postprandial blood glucose; HDL: High-density lipoproteins; LDL: Low-density lipoproteins; VLDL: Very low-density lipoproteins; HbA1c: Glycated hemoglobin.

**Table 3:** Replacement of calories from sugar with stevia-based tabletop sweetener (test product containing steviol glycoside) in the daily diet and estimated daily intake of steviol glycosides (expressed as steviol).

	Group 1	Group 2
Mean amount of sugar ^a^ consumed (g/day) at baseline	26.92 ± 8.05	26.50 ± 5.16
Average amount of calories consumed from sugar (kcal/day) *	107.67	106.00
Average amount of test product ^b^ consumed (g/day)	4.67	4.77
Average amount of calories consumed from excipients ^c^ used in the test product (kcal/day)	16.40	16.70
Calorie deficit by replacing sugar with the test product (kcal/day)	91.27	89.30
EDI of steviol glycoside (active) from the test product (mg/day)	168.17	178.30
Mean weight of subjects at baseline (kg)	70.29 ± 8.15	73.94 ± 7.03
EDI of steviol glycoside (expressed as steviol) (mg/kg body weight/day)	2.39	2.4
ADI of steviol glycoside (expressed as steviol) (mg/kg body weight/day) ^d^	4	4

^a^ Sugar refers to table sugar (sucrose). ^b^ Test product refers to the product containing steviol glycoside in both formats—powder concentrate and pellet. ^c^ Excipients refer to permitted carriers used in the test products, such as simple carbohydrates and sugar alcohols. ^d^ ADI of steviol glycoside (expressed as steviol) as given by JECFA. * Derived values from the mean observation. EDI: Estimated daily intake; ADI: Acceptable daily intake; JECFA: Joint FAO/WHO Expert Committee on Food Additives.

## Data Availability

The data presented in this study are available on request from the corresponding author. The data are not publicly available as they contain information that could compromise the privacy of the research participants.
